# Perception and self-organized instability

**DOI:** 10.3389/fncom.2012.00044

**Published:** 2012-07-06

**Authors:** Karl Friston, Michael Breakspear, Gustavo Deco

**Affiliations:** ^1^The Wellcome Trust Centre for Neuroimaging, University College LondonLondon, UK; ^2^Queensland Institute of Medical Research, Royal Brisbane Hospital, BrisbaneQLD, Australia; ^3^Computational Neuroscience Group, Department of Technology, Universitat Pompeu FabraBarcelona, Spain

**Keywords:** free-energy, self-organization, critical slowing, generalized synchronization, bayesian inference

## Abstract

This paper considers state-dependent dynamics that mediate perception in the brain. In particular, it considers the formal basis of self-organized instabilities that enable perceptual transitions during Bayes-optimal perception. The basic phenomena we consider are perceptual transitions that lead to conscious *ignition* (Dehaene and Changeux, 2011) and how they depend on dynamical instabilities that underlie chaotic itinerancy (Breakspear, 2001; Tsuda, 2001) and self-organized criticality (Beggs and Plenz, 2003; Plenz and Thiagarajan, 2007; Shew et al., 2011). Our approach is based on a dynamical formulation of perception as approximate Bayesian inference, in terms of variational free energy minimization. This formulation suggests that perception has an inherent tendency to induce dynamical instabilities (critical slowing) that enable the brain to respond sensitively to sensory perturbations. We briefly review the dynamics of perception, in terms of generalized Bayesian filtering and free energy minimization, present a formal conjecture about self-organized instability and then test this conjecture, using neuronal (numerical) simulations of perceptual categorization.

## Introduction

Perceptual categorization speaks to two key dynamical phenomena: transitions from one perceptual state to another and the dynamical mechanisms that permit this transition. In terms of perceptual transitions, perception can be regarded as the selection of a single hypothesis from competing alternatives that could explain sensations (Gregory, 1980). This selection necessarily entails a change in the brain's representational or perceptual state—that may be unconscious in the sense of Helmholtz's unconscious inference or conscious. The implicit transition underlies much of empirical neuroscience (for example, event related potentials and brain activation studies) and has been invoked to understand how sensory information “goes beyond unconscious processing and gains access to conscious processing, a transition characterized by the existence of a reportable subjective experience” (Dehaene and Changeux, 2011). Dehaene and Changeux review converging neurophysiological data, acquired during conscious and unconscious processing, that speaks to the neural signatures of conscious access: late amplification of relevant sensory activity, long-distance cortico-cortical synchronization and *ignition* of a large-scale prefronto-parietal network. The notion of ignition calls on several dynamical phenomena that characterize self-organization; such as, distributed processing in coupled non-linear systems, phase transitions and metastability: see also (Fisch, 2009). In what follows, we ask whether the underlying dynamical mechanisms that lead to perceptual transitions and consequent ignition can be derived from basic principles; and, if so, what does this tell us about the self-organized brain.

### Itinerancy and self-organization

One of the most ubiquitous (and paradoxical) dynamical features of self-organizing and *autopoietic* systems (Maturana and Varela, 1980) is their predisposition to destroy their own fixed points. We have referred to this as *autovitiation* to emphasise the crucial role that self-induced instabilities play in maintaining peripatetic or itinerant (wandering) dynamics (Friston, 2010; Friston and Ao, 2012). The importance of itinerancy has been articulated many times in the past (Nara, 2003), particularly from the perspective of computation and autonomy (van Leeuwen, 2008). Itinerancy provides a link between exploration and foraging in ethology (Ishii et al., 2002) and dynamical systems theory approaches to the brain (Freeman, 1994) that emphasise the importance of chaotic itinerancy (Tsuda, 2001) and self-organized critically (Beggs and Plenz, 2003; Pasquale et al., 2008; Shew et al., 2011). Itinerant dynamics also arise from metastability (Jirsa et al., 1994) and underlie important phenomena like winnerless competition (Rabinovich et al., 2008).

The vitiation of fixed points or attractors is a mechanism that appears in several guises and has found important applications in a number of domains. For example, it is closely related to the notion of autopoiesis and self-organization in situated (embodied) cognition (Maturana and Varela, 1980). It is formally related to the destruction of gradients in synergetic treatments of intentionality (Tschacher and Haken, 2007). Mathematically, it finds a powerful application in universal optimization schemes (Tyukin et al., 2003) and, indeed, as a model of perceptual categorization (Tyukin et al., 2009). In what follows, we briefly review the dynamical scenarios that support itinerant dynamics: *chaotic itinerancy*, *heteroclinic cycling* and *multi-stable switching*.

#### Chaotic itinerancy

Chaotic itinerancy refers to the behavior of complicated (usually coupled non-linear) systems that possess weakly attracting sets—*Milnor attractors*—with basins of attraction that are very close to each other. Their proximity destabilises the Milnor attractors to create *attractor ruins*, which allow the system to leave one attractor and explore another, even in the absence of noise. A Milnor attractor is chaotic attractor—onto which the system settles from a set of initial conditions—with positive measure (volume). However, another set of initial conditions (also with positive measure) that belong to the basin of another attractor can be infinitely close; this is called *attractor riddling*. Itinerant orbits typically arise from unstable periodic orbits that reside in (are dense within) the attractor, where the heteroclines of unstable orbits typically connect to another attractor, or they just wander out into state space and then back onto the attractor, giving rise to *bubbling*. In other words, unstable manifolds from saddles densely embedded in the attractors become stable manifolds and connect different attractors. This is a classic scenario for *intermittency*—in which the dynamics are characterized by long laminar (ordered) periods as the system approaches a Milnor attractor and brief turbulent phases, when it gets close to an unstable manifold. If the number of periodic orbits is large, then this can happen indefinitely, because the chaotic Milnor attractor is ergodic. Ergodicity is an important concept and is also a key element of the free energy principle. The term ergodic is used to describe a dynamical system that has the same behavior averaged over time as averaged over its states. The celebrated ergodic theorem is due to Birkhoff (Birkhoff, 1931), and concerns the behavior of systems that have been evolving for a long time: intuitively, an ergodic system forgets its initial states, such that the probability a system is found in any state becomes—for almost every state—the proportion of time that state is occupied. See (Breakspear, 2004) for further discussion and illustrations. See (Namikawa, 2005) for discussion of chaotic itinerancy and power law residence times in attractor ruins.

The notion of Milnor attractors underlies much of the technical and cognitive literature on itinerant dynamics. For example, one can explain “a range of phenomena in biological vision, such as mental rotation, visual search, and the presence of multiple time scales in adaptation” using the concept of weakly attracting sets (Tyukin et al., 2009). The common theme here is the induction of itinerancy through the destabilisation of attracting sets or the gradients causing them (Tschacher and Haken, 2007). The ensuing attractor ruins or relics (Gros, 2009) provide a framework for heteroclinic orbits that are ubiquitous in electrophysiology (Breakspear and Stam, 2005), cognition (Bressler and Tognoli, 2006) and large-scale neuronal dynamics (Werner, 2007).

#### Heteroclinic cycling

In heteroclinic cycling there are no attractors, not even Milnor ones (or at least there is a large open set in state space with no attractors)—only saddles connected one to the other by heteroclinic orbits. A saddle is a point (invariant set) that has both attracting (stable) and repelling (unstable) manifolds. A heteroclinic cycle is a topological circle of saddles connected by heteroclinic orbits. If a heteroclinic cycle is asymptotically stable, the system spends longer and longer periods of time in a neighborhood of successive saddles; producing a peripatetic wandering through state space. The resulting heteroclinic cycles have been proposed as a metaphor for neuronal dynamics that underlie cognitive processing (Rabinovich et al., 2008) and exhibit important behaviors such as winnerless competition, of the sort seen in central pattern generators in the motor system. Heteroclinic cycles have also been used as generative models in the perception of sequences with deep hierarchical structure (Kiebel et al., 2009).

#### Multi-stability and switching

In multistability, there are typically a number of classical attractors—stronger than Milnor attractors in the sense that their basins of attraction not only have positive measure but are also open sets. Open sets are just sets of points that form a neighborhood: in other words, one can move a point in any direction without leaving the set—like the interior of a ball, as opposed to its surface. These attractors are not connected, but rather separated by a basin boundary. However, they are weak in the sense that the basins are shallow (but topologically simple). System noise is then required to drive the system from attractor one to another—this is called *switching*.

Noise plays an obligate role in switching; however, is not a prerequisite for heteroclinic cycling but acts to settle the excursion time around the cycle onto some characteristic time scale. Without noise, the system will gradually slow as it gets closer and closer (but never onto) the cycle. In chaotic itinerancy, the role of noise is determined by the geometry of the instabilities. Multi-stability underlies much of the work on attractor network models of perceptual decisions and categorization; for example, in binocular rivalry (Theodoni et al., 2011).

### Itinerancy and critical slowing

All three scenarios considered above rest on a delicate balance between dynamical stability and instability: chaotic itinerancy requires weakly attracting sets that have unstable manifolds; heteroclinic cycles are based on saddles with unstable manifolds and switching requires classical attractors with shallow basins that can be destabilized by noise. In terms of linear stability analysis, dynamical instability requires the principal Lyapunov exponent—describing the local exponential divergence of flow—to be greater than zero. Generally, when a negative principal Lyapunov exponent approaches zero from below, systems approach a phase transition and exhibit critical slowing. Lyapunov exponents are based on a local linear approximation to flow and describe the rate of exponential decay of small fluctuations about the flow. As the Lyapunov exponents approach zero these fluctuations decay more slowly. However, at some point very near the instability, the local linearization breaks down and higher order non-linear terms from the Taylor series expansion dominate (or at least contribute). At this stage, the system's memory goes from an exponential form to a power law and the fluctuations no longer decay exponentially but can persist, inducing correlations over large distances and timescales. For example, in the brain, long-range cortico-cortical synchronization may be evident over several centimetres and show slow fluctuations (Breakspear et al., 2010). This phenomenon is probably best characterized in continuous phase transitions in statistical physics, where it is referred to as *criticality*. The possibility that critical regimes—in which local Lyapunov exponents fluctuate around zero—are themselves attracting sets leads to the notion of *self-organized criticality* (Bak et al., 1987).

In what follows, *critical slowing* is taken to mean that one or more local Lyapunov exponents approach zero from below. Note that critical slowing does not imply the dynamics *per se* are slow; it means that unstable modes of behavior decay slowly. Indeed, as the principal Lyapunov exponent approaches zero from below, the system can show fast turbulent flow as in intermittency. In what follows, we explore the notion that any self-organizing system that maintains a homoeostatic and ergodic relationship with its environment will tend to show critical slowing. In fact, we will conjecture that critical slowing is mandated by the very processes that underwrite ergodicity. In this sense, the existence of a self-organizing (ergodic) system implies that it will exhibit critical slowing. Put another way, self-organized critical slowing may be a necessary attribute of open ergodic systems.

In the context of self-organized neuronal activity, we will conjecture that perceptual inference mandates critical slowing and is therefore associated with phase transitions and long-range correlations—of the sort that may correspond to the ignition phenomena considered in (Dehaene and Changeux, 2011). So what qualifies the brain as ergodic? Operationally, this simply means that the probability of finding the brain in a particular state is proportional to the number of times that state is visited. In turn, this implies that neuronal states are revisited over sufficiently long periods of time. This fundamental and general form of homoeostasis is precisely what the free energy principle tries to explain.

### Overview

In this paper, we focus on a rather elementary form of self-organized instability; namely the autovitiation of stable dynamics during (Bayes-optimal) perception. In brief, if neuronal activity represents the causes of sensory input, then it should represent uncertainty about those causes in a way that precludes overly confident representations. This means that neuronal responses to stimuli should retain an optimal degree of instability that allows them to explore alternative hypotheses about the causes of those stimuli. To formalise this intuition, we consider neuronal dynamics as performing Bayesian inference about the causes of sensations, using a gradient descent on a (variational free energy) bound on the surprise induced by sensory input. This allows us to examine the stability of this descent in terms of Lyapunov exponents and how local Lyapunov exponents should behave. We will see that the very nature of free energy minimization produces local Lyapunov exponents that fluctuate around small (near zero) values. In other words, Bayes-optimal perception has an inherent tendency to promote critical slowing, which may be necessary for perceptual transitions and consequent categorization.

This paper comprises four sections. The first section reviews Bayes-optimal inference in the setting of free energy minimization to establish the basic imperatives for neuronal activity. In the second section, we look at neuronal implementations of free energy minimization, in terms of predictive coding, and how this relates to the anatomy and physiology of message passing in the brain. In the third section, we consider the dynamics of predictive coding in terms of generalized synchronization and Lyapunov exponents. This section establishes a conjecture that predictive coding will necessarily show self-organized instability. The conjecture is addressed numerically using neuronal simulations of perceptual categorization in the final section. We conclude with a brief discussion of self-organization, over different scales, in relation to the optimality principles on which this approach is based.

## The free energy principle

This section establishes the nature of Bayes-optimal inference in the context of self-organized exchanges with the world. It starts with the basic premise that underlies free energy minimization; namely, the imperative to minimize the dispersion of sensory states to ensure a homoeostasis of the external and internal milieu (Ashby, 1947). We show briefly how action and perception follow from this imperative and highlight the central role of minimizing free energy. This section develops the ideas in a rather compact and formal way. Readers who prefer a nonmathematical description could skip to the summary and discussion of the results at the end of this section.

### Notation and set up

We will use *X* : Ω → ℝ for real valued random variables and *x* ∈ *X* for particular values. A probability density will be denoted by *p*(*x*) = *Pr*{*X* = *x*} using the usual conventions and its entropy *H*[*p*(*x*)] by *H*(*X*). The tilde notation $\tilde{x}=\left(x,x\prime ,x\prime \prime ,\ldots \right)$ denotes variables in generalized coordinates of motion, using the LaGrange notation for temporal derivatives (Friston, 2008). Finally, *E*[·] denotes an expectation or average. For simplicity, constant terms will be omitted from equalities.

In what follows, we would consider free energy minimization in terms of active inference: Active inference rests on the tuple (Ω, Ψ, *S*, *A*, *R*, *q*, *p*) that comprises the following:

- *A sample space* Ω or non-empty set from which random fluctuations or outcomes ω ı Ω are drawn.
- *Hidden states* Ψ : Ψ × *A* × Ω → ℝ that constitute the dynamics of states of the world that cause sensory states and depend on action.
- *Sensory states S* : Ψ × *A* × Ω → ℝ that correspond to the agent's sensations and constitute a probabilistic mapping from action and hidden states.
- *Action A* : *S* × *R* → ℝ that corresponds to action emitted by an agent and depends on its sensory and internal states.
- *Internal states R* : *R* × *S* × Ω → ℝ that constitute the dynamics of states of the agent that cause action and depend on sensory states.
- *Conditional density* $q\left(\tilde{\psi }\right):=q\left(\tilde{\psi }|\tilde{\mu }\right)$—an arbitrary probability density function over hidden states $\tilde{\psi }\in \psi$ that is parameterized by internal states $\tilde{\mu }\in R$.
- *Generative density* $p\left(\tilde{s},\tilde{\psi }|m\right)$—a probability density function over external (sensory and hidden) states under a generative model denoted by *m*. This model specifies the Gibbs energy of any external states: $G\left(\tilde{s},\tilde{\psi }\right)=-\text{In }p\left(\tilde{s},\tilde{\psi }|m\right)$.

We assume that the imperative for any biological system is to minimize the dispersion of its sensory states, with respect to action: mathematically, this dispersion corresponds to the (Shannon) entropy of the probability density over sensory states. Under ergodic assumptions, this entropy is equal to the long-term time average of surprise (almost surely):

(1) $$\begin{array}{c} H(S)=E_{t}[ℒ(\tilde{s}(t))]\\ ℒ=-\text{In }p(\tilde{s}(t)|m) \end{array}$$

Surprise (or more formally surprisal or self information) $ℒ\left(\tilde{s}\right)$ is defined by the generative density or model. This means that the entropy of sensory states can be minimized through action

(2) $$a\left(t\right)=\underset{a\;\in \;A}{\text{argmin}}\left\{ℒ\left(\tilde{s}\left(t\right)\right)\right\}$$

When Equation (2) is satisfied, the variation of entropy in Equation (1) with respect to action is zero, which means sensory entropy has been minimized (at least locally). From a statistical perspective, surprise is called negative log evidence, which means that minimizing surprise is the same as maximizing the Bayesian model evidence for the agent's generative model.

### Action and perception

Action cannot minimize sensory surprise directly (Equation 2) because this would involve an intractable marginalization over hidden states (an impossible averaging over all hidden states to obtain the probability density over sensory states)—so surprise is replaced with an upper bound called variational free energy (Feynman, 1972). This free energy is a functional of the conditional density or a function of the internal states that parameterise the conditional density. The conditional density is a key concept in inference and is a probabilistic representation of the unknown or hidden states. It is also referred to as the recognition density. Unlike surprise, free energy can be quantified because it depends only on sensory states and the internal states that parameterise the conditional density. However, replacing surprise with free energy means that internal states also have to minimize free energy, to ensure it is a tight bound on surprise:

(3) $$\begin{array}{c} a\left(t\right)=\underset{a\;\in \;A}{\text{argmin}}\left\{F\left(\tilde{s}\left(t\right),\tilde{\mu }\left(t\right)\right)\right\}\\ \tilde{\mu }\left(t\right)=\underset{\tilde{\mu }\;\in \;R}{\text{argmin}}\left\{F\left(\tilde{s}\left(t\right),\tilde{\mu }\right)\right\}\\ F=E_{q}\left[G\left(\tilde{s},\tilde{\psi }\right)\right]-H\left[q\left(\tilde{\psi }|\tilde{\mu }\right)\right]\\ =ℒ\left(\tilde{s}\right)+D\left[q\left(\tilde{\psi }\right)||p\left(\tilde{\psi }|\tilde{s},m\right)\right]\geq ℒ\left(\tilde{s}\right) \end{array}$$

This induces a dual minimization with respect to action and the internal states. These minimizations correspond to *action* and *perception* respectively. In brief, the need for perception is induced by introducing free energy to finesse the evaluation of surprise; where free energy can be evaluated by an agent fairly easily, given a Gibbs energy or generative model. Gibbs energy is just the surprise or improbability associated with a combination of sensory and hidden states. This provides a probabilistic specification of how sensory states are generated from hidden states. The last equality above says that free energy is always greater than surprise because the second (Kullback-Leibler divergence) term is non-negative. This means that when free energy is minimized with respect to the internal states, free energy approximates surprise and the conditional density approximates the posterior density over hidden states:

(4) $$D\left[q\left(\tilde{\psi }\right)||p\left(\tilde{\psi }|\tilde{s},m\right)\right]\approx 0\Rightarrow \left\{ \begin{array}{l} F\left(\tilde{s},\tilde{\mu }\right)\approx ℒ\left(\tilde{s}\right)\\ \;\;\;\;\;q\left(\tilde{\psi }\right)\approx p\left(\tilde{\psi }|\tilde{s},m\right) \end{array}\right.$$

This is known as approximate Bayesian inference, which becomes exact when the conditional and posterior densities have the same form (Beal, 2003). The only outstanding issue is the form of the conditional density adopted by an agent:

### The maximum entropy principle and the Laplace assumption

If we admit an encoding of the conditional density up to second order moments, then the maximum entropy principle (Jaynes, 1957) implicit in the definition of free energy (Equation 3) requires $q\left(\tilde{\psi }|\tilde{\mu }\right)=N\left(\tilde{\mu },\Sigma \right)$ to be Gaussian. This is because a Gaussian density has the maximum entropy of all forms that can be specified with two moments. Assuming a Gaussian form is known as the *Laplace assumption* and enables us to express the entropy of the conditional density in terms of its first moment or expectation. This follows because we can minimize free energy with respect to the conditional covariance as follows:

(5) $$\begin{array}{c} F=G(\tilde{s},\tilde{\mu })+\frac{1}{2}tr(\Sigma \cdot \partial_{\tilde{\mu }\tilde{\mu }}G)-\frac{1}{2}\text{in}|\Sigma |\\ \Rightarrow \partial_{\Sigma }F=\frac{1}{2}\partial_{\tilde{\mu }\tilde{\mu }}G-\frac{1}{2}\Pi \\ \partial_{\Sigma }F=0\Rightarrow \left\{ \begin{array}{l} \Pi =\partial_{\tilde{\mu }\tilde{\mu }}G\\ F=G(\tilde{s},\tilde{\mu })+\frac{1}{2}\text{In}|\partial_{\tilde{\mu }\tilde{\mu }}G| \end{array}\right. \end{array}$$

Here, the conditional precision $\Pi \left(\tilde{s},\tilde{\mu }\right)$ is the inverse of the conditional covariance $\Sigma \left(\tilde{s},\tilde{\mu }\right)$. In short, free energy is a function of the conditional expectations (internal states) and sensory states.

### Summary

**Figure 1:**
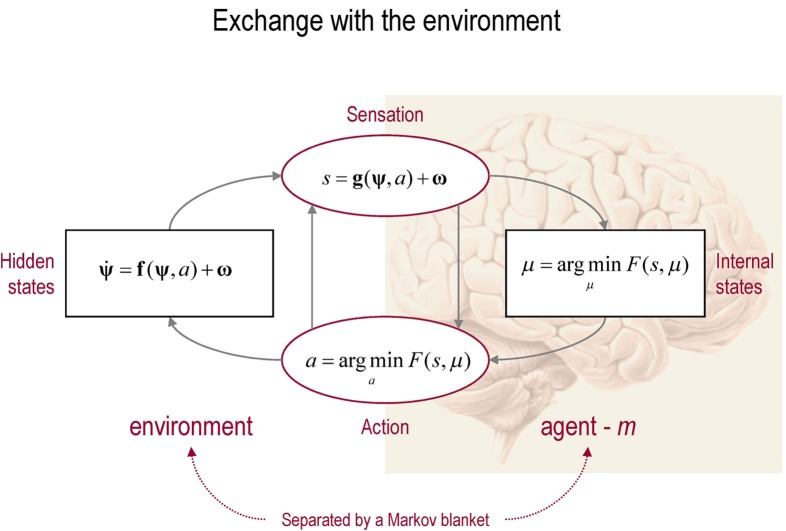
**This schematic shows the dependencies among various quantities modelling exchanges of a self-organizing system like the brain with the environment.** It shows the states of the environment and the system in terms of a probabilistic dependency graph, where connections denote directed dependencies. The quantities are described within the nodes of this graph, with exemplar forms for their dependencies on other variables (see main text). Here, hidden and internal states are separated by action and sensory states. Both action and internal states encoding a conditional density minimize free energy. Note that hidden states in the real world and the form of their dynamics are different from that assumed by the generative model; this is why hidden states are in bold. See main text for details.

To recap, we started with the assumption that biological systems minimize the dispersion or entropy of sensory states to ensure a sustainable and homoeostatic exchange with their environment (Ashby, 1947). Clearly, this entropy cannot be measured or changed directly. However, if agents know how their action changes sensations (for example, if they know contracting certain muscle fibres will necessarily excite primary sensory afferents from stretch receptors), then they can minimize the dispersion of their sensory states by countering surprising deviations from their predictions. Minimizing surprise through action is not as straightforward as it might seem, because surprise *per se* is an intractable quantity to estimate. This is where free energy comes in—to provide an upper bound that enables agents to minimize free energy instead of surprise. However, in creating the upper bound, the agent now has to minimize the difference between surprise and free energy by changing its internal states. This corresponds to perception and makes the conditional density an approximation to the true posterior density in a Bayesian sense (Helmholtz, 1866/1962; Gregory, 1980; Ballard et al., 1983; Dayan et al., 1995; Friston, 2005; Yuille and Kersten, 2006). See Figure 1 for a schematic summary. We now turn to neurobiological implementations of this scheme, with a special focus on hierarchical message passing in the brain and the associated neuronal dynamics.

## Neurobiological implementation of active inference

In this section, we take the general principles above and consider how they might be implemented in the brain. The equations in this section may appear a bit complicated; however, they are based on just three assumptions:

- The brain minimizes the free energy of sensory inputs defined by a generative model.
- The generative model used by the brain is hierarchical, non-linear and dynamic.
- Neuronal firing rates encode the expected state of the world, under this model.

The first assumption is the free energy principle, which leads to active inference in the embodied context of action. The second assumption is motivated easily by noting that the world is both dynamic and non-linear and that hierarchical causal structure emerges inevitably from a separation of temporal scales (Ginzburg and Landau, 1950; Haken, 1983). The final assumption is the Laplace assumption that, in terms of neural codes, leads to the *Laplace code*, which is arguably the simplest and most flexible of all neural codes (Friston, 2009).

Given these assumptions, one can simulate a whole variety of neuronal processes by specifying the particular equations that constitute the brain's generative model. The resulting perception and action are specified completely by the above assumptions and can be implemented in a biologically plausible way as described below (see Table 1 for a list of previous applications of this scheme). In brief, these simulations use differential equations that minimize the free energy of sensory input using a generalized gradient descent (Friston et al., 2010).

(6) $$\begin{array}{c} \dot{\tilde{\mu }}\left(t\right)=D\tilde{\mu }\left(t\right)-\partial_{\tilde{\mu }}F\left(\tilde{s},\tilde{\mu }\right)\\ \dot{a}\left(t\right)=-\partial_{a}F\left(\tilde{s},\tilde{\mu }\right) \end{array}$$

**Table 1:** **Processes and paradigms that have been modelled using generalized filtering**.

**Domain**	**Process or paradigm**
Perception	Perceptual categorization (bird songs) (Friston and Kiebel, 2009a) Novelty and omission-related responses (Friston and Kiebel, 2009b)
	Perceptual inference (speech) (Kiebel et al., 2009)
Illusions	The Cornsweet illusion and Mach bands (Brown and Friston, 2012)
Sensory learning	Perceptual learning (mismatch negativity) (Friston and Kiebel, 2009a,b)
Attention	Attention and the Posner paradigm (Feldman and Friston, 2010) Attention and biased competition (Feldman and Friston, 2010)
Motor control	Retinal stabilization and oculomotor reflexes (Friston et al., 2010) Orienting and cued reaching (Friston et al., 2010) Motor trajectories and place cells (Friston et al., 2011)
Sensorimotor integration	Bayes-optimal sensorimotor integration (Friston et al., 2010)
Visual search	Saccadic eye movements (Friston et al., 2012)
Behavior	Heuristics and dynamical systems theory (Friston and Ao, 2012) Goal-directed behavior (Friston et al., 2009)
Action observation	Action observation and mirror neurons (Friston et al., 2011)
Action selection	Affordance and sequential behavior (Friston et al., 2012)

These coupled differential equations describe perception and action respectively and just say that internal brain states and action change in the direction that reduces free energy. The first is known as (generalized) predictive coding and has the same form as Bayesian (e.g., Kalman-Bucy) filters used in time series analysis; see also (Rao and Ballard, 1999). The first term in Equation (6) is a prediction based upon a matrix differential operator D that returns the generalized motion of conditional expectations, such that $D\tilde{\mu }=\left(\mu \prime ,\mu \prime \prime ,\mu \prime \prime ,\ldots \right)$. The second term is usually expressed as a mixture of prediction errors that ensures the changes in conditional expectations are Bayes-optimal predictions about hidden states of the world. The second differential equation says that action also minimizes free energy. The differential equations are coupled because sensory input depends upon action, which depends upon perception through the conditional expectations. This circular dependency leads to a sampling of sensory input that is both predicted and predictable, thereby minimizing free energy and surprise.

To perform neuronal simulations using this generalized descent, it is only necessary to integrate or solve Equation (6) to simulate neuronal dynamics that encode the conditional expectations and ensuing action. Conditional expectations depend upon the brain's generative model of the world, which we assume has the following (hierarchical) form

(7) $$\begin{array}{c} s=g^{\left(1\right)}\left(x^{\left(1\right)},v^{\left(1\right)},u^{\left(1\right)}\right)+\omega_{v}^{\left(1\right)}\\ {\dot{x}}^{\left(1\right)}=f^{\left(1\right)}\left(x^{\left(1\right)},v^{\left(1\right)},u^{\left(1\right)}\right)+\omega_{x}^{\left(1\right)}\\ \vdots \\ v^{\left(i-1\right)}=g^{\left(i\right)}\left(x^{\left(i\right)},v^{\left(i\right)},u^{\left(i\right)}\right)+\omega_{v}^{\left(i\right)}\\ {\dot{x}}^{\left(i\right)}=f^{\left(i\right)}\left(x^{\left(i\right)},v^{\left(i\right)},u^{\left(i\right)}\right)+\omega_{x}^{\left(i\right)}\\ \vdots \end{array}$$

This equation is just a way of writing down a model that specifies the generative density over the sensory and hidden states, where the hidden states Ψ = *X* × *V* have been divided into hidden dynamic states and causes. Here, (*g*^(*i*)^,*f*^(*i*)^) are non-linear functions of hidden states that generate sensory inputs at the first (lowest) level, where for notational convenience, *v*^(0)^ := s.

Hidden causes *V* ⊂ Ψ can be regarded as functions of hidden dynamic states; hereafter, hidden states *X*⊂Ψ. Random fluctuations (ω^(*i*)^_*x*_, ω^(*i*)^_v_) on the motion of hidden states and causes are conditionally independent and enter each level of the hierarchy. It is these that make the model probabilistic—they play the role of sensory noise at the first level and induce uncertainty about states at higher levels. The (inverse) amplitudes of these random fluctuations are quantified by their precisions (Π^(*i*)^_*x*_, Π^(*i*)^_*v*_), which we assume to be fixed in this paper (but see conclusion). Hidden causes link hierarchical levels, whereas hidden states link dynamics over time. Hidden states and causes are abstract quantities that the brain uses to explain or predict sensations (like the motion of an object in the field of view). In hierarchical models of this sort, the output of one level acts as an input to the next. This input can produce complicated (generalized) convolutions with deep (hierarchical) structure.

### Perception and predictive coding

Given the form of the generative model (Equation 7) we can now write down the differential equations (Equation 6) describing neuronal dynamics in terms of (precision-weighted) prediction errors on the hidden causes and states. These errors represent the difference between conditional expectations and predicted values, under the generative model (using *A* · *B* := *A*^*T*^ *B* and omitting higher-order terms):

(8) $$\begin{array}{c} {\dot{\tilde{\mu }}}_{x}^{\left(i\right)}=D{\tilde{\mu }}_{x}^{\left(i\right)}+\frac{\partial {\tilde{g}}^{\left(i\right)}}{\partial {\tilde{\mu }}_{x}^{\left(i\right)}}\cdot \xi_{v}^{\left(i\right)}+\frac{\partial {\tilde{f}}^{\left(i\right)}}{\partial {\tilde{\mu }}_{x}^{\left(i\right)}}\cdot \xi_{v}^{\left(i\right)}-D^{T}\xi_{x}^{\left(i\right)}\\ {\dot{\tilde{\mu }}}_{v}^{\left(i\right)}=D{\tilde{\mu }}_{v}^{\left(i\right)}+\frac{\partial {\tilde{g}}^{\left(i\right)}}{\partial {\tilde{\mu }}_{v}^{\left(i\right)}}\cdot \xi_{v}^{\left(i\right)}+\frac{\partial {\tilde{f}}^{\left(i\right)T}}{\partial {\tilde{\mu }}_{v}^{\left(i\right)}}\cdot \xi_{v}^{\left(i\right)}-\xi_{v}^{\left(i+1\right)}\\ \xi_{x}^{\left(i\right)}=\Pi_{x}^{\left(i\right)}\left(D{\tilde{\mu }}_{x}^{\left(i\right)}-{\tilde{f}}^{\left(i\right)}\left({\tilde{\mu }}_{x}^{\left(i\right)},{\tilde{\mu }}_{v}^{\left(i\right)}\right)\right)\\ \xi_{v}^{\left(i\right)}=\Pi_{v}^{\left(i\right)}\left({\tilde{\mu }}_{v}^{\left(i-1\right)}-{\tilde{g}}^{\left(i\right)}\left({\tilde{\mu }}_{x}^{\left(i\right)},{\tilde{\mu }}_{v}^{\left(i\right)}\right)\right) \end{array}$$

Equation (8) can be derived fairly easily by computing the free energy for the hierarchical model in Equation (7) and inserting its gradients into Equation (6). This gives a relatively simple update scheme, in which conditional expectations are driven by a mixture of prediction errors, where prediction errors are defined by the equations of the generative model.

**Figure 2:**
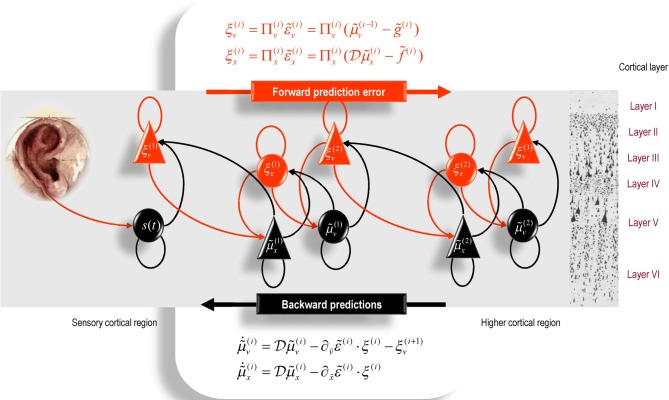
**Schematic detailing a neuronal architecture that might encode conditional expectations about the states of a hierarchical model.** This shows the speculative cells of origin of forward driving connections that convey prediction error from a lower area to a higher area and the backward connections that construct predictions (Mumford, 1992). These predictions try to explain away prediction error in lower levels. In this scheme, the sources of forward and backward connections are superficial and deep pyramidal cells respectively. The equations represent a generalized descent on free energy under the hierarchical model described in the main text: see also (Friston, 2008). State-units are in black and error-units in red. Here, neuronal populations are deployed hierarchically within three cortical areas (or macro-columns). Within each area, the cells are shown in relation to cortical layers: supra-granular (I–III) granular (IV) and infra-granular (V–VI) layers.

It is difficult to overstate the generality and importance of Equation (8): its solutions grandfather nearly every known statistical estimation scheme, under parametric assumptions about additive or multiplicative noise (Friston, 2008). These range from ordinary least squares to advanced variational deconvolution schemes. The resulting scheme is called *generalized filtering* or predictive coding (Friston et al., 2010). In neural network terms, Equation (8) says that error-units receive predictions from the same level and the level above. Conversely, conditional expectations (encoded by the activity of state units) are driven by prediction errors from the same level and the level below. These constitute bottom-up and lateral messages that drive conditional expectations toward a better prediction to reduce the prediction error in the level below. This is the essence of recurrent message passing between hierarchical levels to optimize free energy or suppress prediction error: see (Friston and Kiebel, 2009a) for a more detailed discussion. In neurobiological implementations of this scheme, the sources of bottom-up prediction errors, in the cortex, are thought to be superficial pyramidal cells that send forward connections to higher cortical areas. Conversely, predictions are conveyed from deep pyramidal cells, by backward connections, to target (polysynaptically) the superficial pyramidal cells encoding prediction error (Mumford, 1992; Friston and Kiebel, 2009a,b). Figure 2 provides a schematic of the proposed message passing among hierarchically deployed cortical areas. Although this paper focuses on perception, for completeness we conclude this section by looking at the neurobiology of action.

### Action

In active inference, conditional expectations elicit behavior by sending top-down predictions down the hierarchy that are unpacked into proprioceptive predictions at the level of the cranial nerve nuclei and spinal-cord. These engage classical reflex arcs to suppress proprioceptive prediction errors and produce the predicted motor trajectory

(9) $$\dot{a}=-\frac{\partial }{\partial a}F=-\frac{\partial \tilde{s}}{\partial a}\cdot \xi_{v}^{\left(1\right)}$$

The reduction of action to classical reflexes follows because the only way that action can minimize free energy is to change sensory (proprioceptive) prediction errors by changing sensory signals; cf., the equilibrium point formulation of motor control (Feldman and Levin, 1995). In short, active inference can be regarded as equipping a generalized predictive coding scheme with classical reflex arcs: see (Friston et al., 2009, 2010) for details. The actual movements produced clearly depend upon top-down predictions that can have a rich and complex structure, due to perceptual optimization based on the sampling of salient exteroceptive and interoceptive inputs.

### Summary

In summary, we have derived equations for the dynamics of perception and action using a free energy formulation of adaptive (Bayes-optimal) exchanges with the world and a generative model that is both generic and biologically plausible. Intuitively, all we have done is to apply the principle of free energy minimization to a particular model of how sensory inputs are caused. This model is called a generative model because it can be used to generate sensory samples and thereby predict sensory inputs for any given set of hidden states. By requiring hidden states to minimize free energy, they become Bayes-optimal estimates of hidden states in the real world—because they implicitly maximize Bayesian model evidence. One simple scheme—that implements this minimization—is called predictive coding and emerges when random effects can be modelled as additive Gaussian fluctuations. Predictive coding provides a neurobiological plausible scheme for inferring states of the world that reduces, essentially, to minimizing prediction errors; namely, the difference between what is predicted—given the current estimates of hidden states—and the sensory inputs actually sampled.

In what follows, we use Equations (6), (7), and (8) to treat neuronal responses in terms of predictive coding. A technical treatment of the material above will be found in (Friston et al., 2010), which provides the details of the generalized descent or filtering used to produce the simulations in the last section. Before looking at these simulations, we consider the nature of generalized filtering and highlight its curious but entirely sensible dynamical properties.

## Self-organized instability

This section examines self-organization in the light of minimizing free energy. These arguments do not depend in any specific way on predictive coding or the neuronal implementation of free energy minimization—they apply to any self-organizing system that minimizes the entropy of the (sensory) states that drive its internal states; either exactly by minimizing (sensory) surprise or approximately by minimizing free energy. In what follows, we will first look at the basic form of the dynamics implied by exposing a self-organizing system to sensory input in terms of skew product systems. A skew product system comprises two coupled systems, where the states of one system influence the flow of states in the other—in our case, hidden states in the world influence neuronal dynamics. These coupled systems invoke the notion of (generalized) synchronization as quantified by conditional Lyapunov exponents (CLE). This is important because the dynamics of a generalized descent on free energy have some particular implications for the CLE. These implications allow us to conjecture that the local Lyapunov exponents will fluctuate around small (near zero) values, which is precisely the condition for chaotic itinerancy and critical slowing. By virtue of the fact that this critical slowing is self-organized, it represents an elementary form of self-organized criticality; namely self-organized critical slowing. In the next section, we will test this conjecture numerically with simulations of perception, using the predictive coding scheme of the previous section.

### Conditional Lyapunov exponents and generalized synchrony

Conditional Lyapunov exponents are normally invoked to understand synchronization between two systems that are coupled, usually in a unidirectional manner, so that there is a *drive* (or master) system and a *response* (or slave) system. The conditional exponents are those of the response system, where the drive system is treated as a source of a (chaotic) drive. Synchronization of chaos is often understood as a behavior in which two coupled systems exhibit identical chaotic oscillations—referred to as identical synchronization (Hunt et al., 1997; Barreto et al., 2003). The notion of chaotic synchronization has been generalized for coupled non-identical systems with unidirectional coupling or a *skew product structure* (Pyragas, 1997):

(10) $$\begin{array}{c} \dot{\tilde{\psi }}=G_{\psi }\left(\tilde{\psi }\right)\\ \dot{\tilde{\mu }}=G_{R}\left(\tilde{\mu },\tilde{\psi }\right) \end{array}$$

Crucially, if we ignore action, neuronal dynamics underlying perception have this skew product structure, where $G\psi \left(\tilde{\psi }\right)$ corresponds to the flow of hidden states and $G_{R}=D\tilde{\mu }-\partial_{\tilde{\mu }}F\left(\tilde{s}\left(\tilde{\psi }\right),\tilde{\mu }\right)$ corresponds to the dynamical response. This is important because it means one can characterize the coupling of hidden states in the world to self-organized neuronal responses, in terms of generalized synchronization.

Generalized synchronization occurs if there exists a map Φ : Ψ → *R* from the trajectories of the (random) attractor in the driving space to the trajectories of the response space, such that $\tilde{\mu }\left(t\right)=\Phi \left(\tilde{\psi }\left(t\right)\right)$. Depending on the properties of the map Φ : Ψ → R, generalized synchronization can be of two types: weak and strong. Weak synchronization is associated with a continuous *C*^0^ but non-smooth map, where the synchronization manifold *M* = {(Ψ, R) : Φ (Ψ) = R} has a fractal structure and the dimension *D*_Ψ× R_ of the attractor in the full state space Ψ × *R* is larger than the dimension of the attractor *D*_Ψ_ in the driving Ψ subspace—that is *D*_Ψ× *R*_ > *D*_Ψ_.

Strong synchronization implies a smooth map (*C*^1^ or higher) and arises when the response system does not inflate the global dimension, *D*_Ψ× *R*_ = *D*_Ψ_. This occurs with identical synchronization, which is a particular case Φ(Ψ) = Ψ of strong synchronization. The global and driving dimensions can be estimated from the appropriate Lyapunov exponents λ_1_ ≥ λ_2_ ≥ … using the Kaplan-Yorke conjecture (Kaplan and Yorke, 1979)

(11) $$D=k+\sum_{i=1}^{k}\frac{\lambda_{i}}{\left|\lambda_{k+1}\right|}$$

Here, λ_1_ ≥ … ≥ λ_*k*_ are the *k* largest exponents for which the sum is non-negative. Strong synchronization requires the principal Lyapunov exponent of the response system (neuronal dynamics) to be less than the *k*-th Lyapunov exponent of the driving system (the world), while weak synchronization just requires it to be less than zero.

The Lyapunov exponents of a dynamical system characterize the rate of separation of infinitesimally close trajectories and provide a measure of contraction or expansion of the state space occupied. For our purposes, they can be considered the eigenvalues of the Jacobian that describes the rate of change of flow, with respect to the states. The *global Lyapunov exponents* correspond to the long-term time average of *local Lyapunov exponents* evaluated on the attractor (the existence of this long-term average is guaranteed by Oseledets theorem). Lyapunov exponents also determine the stability or instability of the dynamics, where negative Lyapunov exponents guarantee Lyapunov stability (of the sort associated with fixed point attractors). Conversely, one or more positive Lyapunov exponents imply (local) instability and (global) chaos. Any (negative) Lyapunov exponent can also be interpreted as the rate of decay of the associated eigenfunction of states, usually referred to as (Oseledets) *modes*. This means as a (negative) Lyapunov exponent approaches zero from below, perturbations of the associated mode decay more slowly. We will return to this interpretation of Lyapunov exponents in the context of stability later. For skew product systems, the CLE correspond to the eigenvalues of the Jacobian $\partial_{\tilde{\mu }}G_{R}\left(\tilde{\mu },\tilde{\psi }\right)$ mapping small variations in the internal states to their motion.

### Critical slowing and conditional Lyapunov exponents

This characterization of coupled dynamical systems means that we can consider the brain as being driven by sensory fluctuations from the environment. The resulting skew product system suggests that neuronal dynamics should show weak synchronization with the sensorium, which means that the maximal (principal) conditional Lyapunov exponent should be less than zero. However, if neuronal dynamics are generating predictions, by modelling the causes of sensations, then these dynamics should themselves be chaotic—because the sensations are caused by itinerant dynamics in the world. So, how can generalized synchronization support chaotic dynamics when the principal CLE is negative?

**Figure 3:**
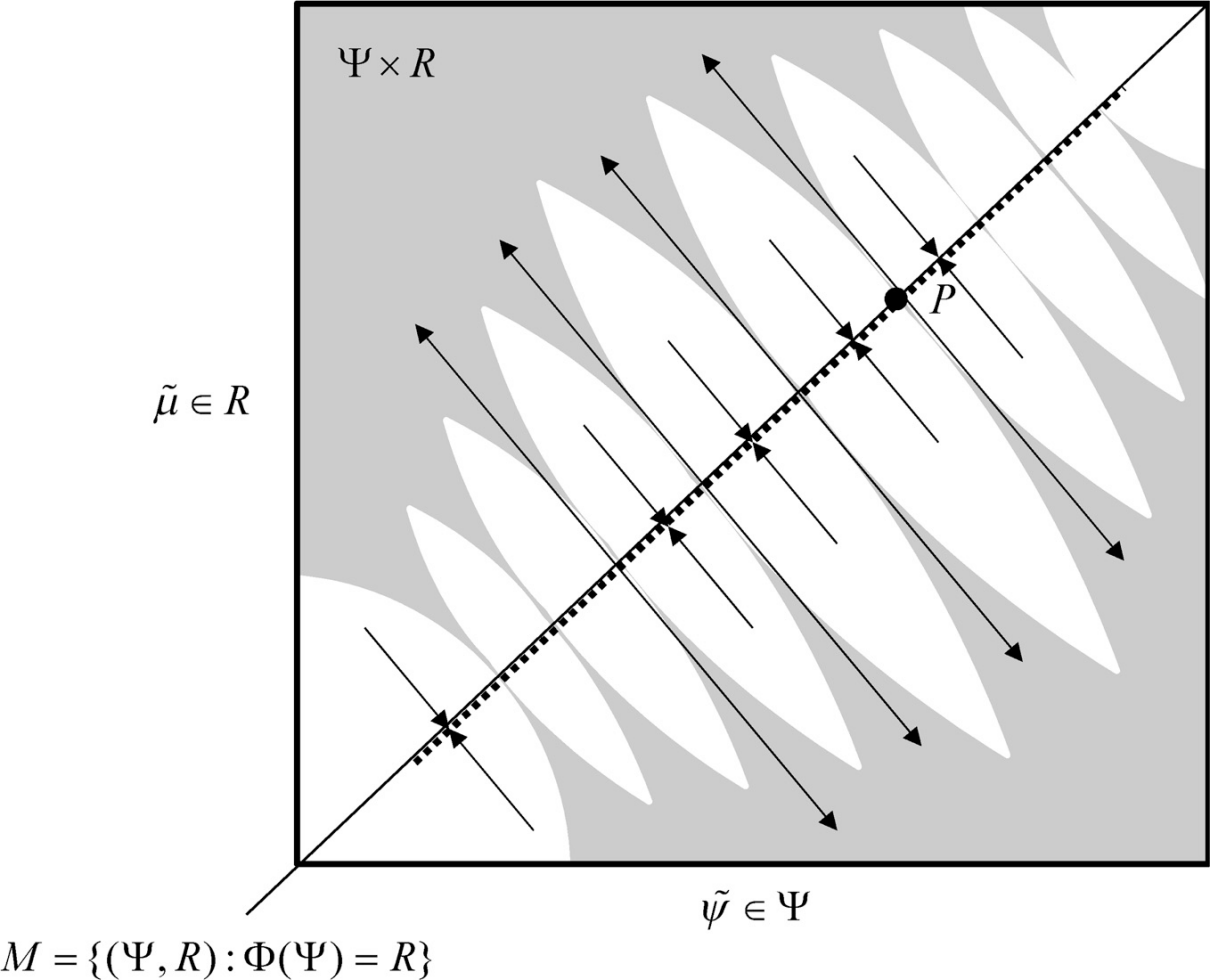
**Schematic representation of synchronization manifold with weak transverse stability—adapted from (Breakspear, 2001):** A Milnor attractor (dotted line) is contained with a synchronization manifold—here an identity mapping. Unstable saddle points such as *P* are repelling in the transverse direction and create narrow tongues of repelling regions (grey regions). Other orbits are attracted toward the chaotic attractor contained within the synchronization manifold.

In skew product systems of the sort above it is useful to partition the Lyapunov exponents into those pertaining to *tangential* flow within the synchronization manifold and *transverse* flow away from the manifold (Breakspear, 2004). In the full state space, the tangential Lyapunov exponents can be positive such that the motion on the synchronization manifold is chaotic, as in the driving system, while the transverse Lyapunov exponents are negative (or close to zero) so that the response system is weakly synchronized with the drive system. See Figure 3 for a schematic illustration of tangential and transverse stability. In short, negative transverse Lyapunov exponents ensure the synchronization manifold *M* ⊂ Ψ × *R* is transversely stable or (equivalently) negative CLE ensure the synchronized manifold *R* = Φ(Ψ) is stable (Pyragas, 1997). In the present setting, this means that the sensorium enslaves chaotic neuronal responses. See (Breakspear, 2001) for a treatment of chaotic itinerancy and generalized synchronization as the basis of olfactory perception: By studying networks of Milnor attractors (Breakspear, 2001) shows how different sensory perturbations can evoke specific switches between various patterns of activity.

Although generalized synchronization provides a compelling metaphor for perception, it also presents a paradox: if the CLE are negative and the synchronized manifold is stable, there is no opportunity for neuronal dynamics (conditional expectations) to jump to another attractor and explore alternative hypotheses. This dialectic is also seen in system identification, where the synchronization between an observed dynamical system and a model system is used to optimize model parameters by maximizing synchronization. However, if the coupling between the observations and the model is too strong, the variation of synchronization with respect to the parameters is too small to permit optimization. This leads to the notion of *balanced synchronization* that requires the CLE “remain negative but small in magnitude” (Abarbanel et al., 2008). In other words, we want the synchronization between the causes of sensory input and neuronal representations to be strong but not too strong. Here, we resolve this general dialectic with the conjecture that Bayes-optimal synchronization is inherently balanced:

#### Conjecture

Dynamical systems that minimize variational free energy dynamically show self-organized critical slowing, with local CLE λ(*t*) ∈ ℝ that fluctuate around small (near zero) values, where

(12) $$\begin{array}{c} \lambda =\text{eig}\left(\partial_{\tilde{\mu }}G_{R}\right)\\ G_{R}=D\tilde{\mu }-\partial_{\tilde{\mu }}F\left(\tilde{s}\left(\psi \right),\tilde{\mu }\right) \end{array}$$

#### Proof

From Equation (6), one can see that the Jacobian can be decomposed into prediction and update terms

(13) $$\partial_{\tilde{\mu }}G_{R}=D-\partial_{\tilde{\mu }\tilde{\mu }}F\left(\tilde{s}\left(t\right),\tilde{\mu }\left(t\right)\right)$$

The contribution of the second (update) depends upon the curvature of the variational free energy. This will always have negative eigenvalues, because the curvature is positive definite. Conversely, the first (prediction) term has eigenvalues of zero. This means, as the free energy curvature decreases the eigenvalues of the Jacobian will get smaller (and can indeed become positive for small but finite curvatures). This is important for two reasons; first, because the free energy changes with time, the local CLE will fluctuate. Second, because the system is minimizing free energy, it is implicitly minimizing the curvature (conditional precision) and is therefore driving some local CLE toward zero (and possibly positive) values. In short, free energy minimization will tend to produce local CLE that fluctuate at near zero values and exhibit self-organized instability or slowing. More formally:

Let 0 ≤ γ_1_ ≤ γ_2_ ≤ … be the real valued positive eigenvalues of the curvature of Gibbs energy or conditional precision. From Equation (5), the free energy can be expressed in terms of these Gibbs exponents

(14) $$\begin{array}{l} F=G+\frac{1}{2}\text{In}|\partial_{\tilde{\mu }\tilde{\mu }}G|\\ =G+\frac{1}{2}\sum_{i}\text{In}\gamma_{i} \end{array}\Rightarrow \frac{\partial F}{\partial \gamma_{i}}=\frac{1}{2\gamma_{i}}\geq 0$$

This shows that the greatest contribution (In γ_1_ « 0) to free energy comes from the smallest exponent—and changes in free energy, with respect to the Gibbs exponents, are greater for smaller values. Therefore, all other things being equal, a generalized descent on free energy will reduce small Gibbs exponents toward zero.

So how are the Lyapunov and Gibbs exponents related? By ignoring third and higher derivatives of Gibbs energy, we can approximate the curvature of the free energy with the curvature of the Gibbs energy: From Equations (5) and (13)

(15) $$\begin{array}{c} \partial_{\tilde{\mu }\tilde{\mu }}F=\partial_{\tilde{\mu }\tilde{\mu }}G+\partial_{\tilde{\mu }\tilde{\mu }}\frac{1}{2}\text{In|}\partial_{\tilde{\mu }\tilde{\mu }}G|\\ \Rightarrow \\ \partial_{\tilde{\mu }}G_{R}\approx D-\partial_{\tilde{\mu }\tilde{\mu }}G \end{array}$$

The relationship between the Lyapunov exponents (eigenvalues of $D-\partial_{\tilde{\mu }\tilde{\mu }}G$) and Gibbs exponents (eigenvalues of $\partial_{\tilde{\mu }\tilde{\mu }}G$) is not simple; however, if we assume that $\partial_{\tilde{\mu }\tilde{\mu }}G$ is approximately diagonal then

(16) $$\lambda =\text{eig}\left(D-\partial_{\tilde{\mu }\tilde{\mu }}G\right)\approx \text{eig}\left(-\partial_{\tilde{\mu }\tilde{\mu }}G\right)=-\gamma$$

In other words, the Lyapunov exponents approximate the negative Gibbs exponents. This means that a generalized descent on free energy will be attracted to inherently unstable minima, with a low curvature and small local CLE. ■

We could motivate the diagonal approximation of the curvature above by noting diagonal forms of the conditional covariance minimize free energy. However, off-diagonal terms are usually quite pronounced and indicate conditional dependencies among representations. The associated off-diagonal terms in the curvature mean that λ ≈ −γ only holds for large exponents, while small Lyapunov exponents are greater than their corresponding (negative) Gibbs exponents. This means that a generalized descent on free energy can become transiently chaotic with positive Lyapunov exponents. We will see an example of this later.

Heuristically, this self-organized instability follows from the principle of maximum entropy (that generalises Laplace's principle of indifference) and reflects the intuition that, while being faithfully responsive to sensory information, it is important to avoid very precise and particular interpretations. From a dynamical perspective, it implies an active maintenance of critically slow (Oseledets) modes, whose CLE are close to zero. In summary, dynamical (approximate) Bayesian inference schemes are inherently self-destabilizing because they search out explanations for data that have the largest margin of error (smallest conditional precision). This produces instability and a critical slowing of the implicit gradient descent. In the next section, we will use a heuristic measure of this slowing:

(17) $$C=\sum_{i}\exp\left(\tau \cdot \lambda_{i}\right)$$

This is simply a sum of the exponential CLE that discounts large negative values. It can be thought of, roughly, as the number of small CLE, where smallness is controlled by a scale parameter τ. Alternatively, the components of the sum in Equation (17) can be regarded as the relative amplitude of a perturbation to the associated mode after τ units of time. In systems with a large number of small negative CLE, these relative amplitudes will be preserved and critical slowing will be large. For systems that show generalized synchronization (where all the CLE are negative) the critical slowing in Equation (17) is upper bounded by the number of CLE.

### Summary

In summary, we have reviewed the central role of Lyapunov exponents in characterizing dynamics; particularly in the context of generalized (weak or strong) synchronization. This is relevant from the point of view of neuronal dynamics, because we can cast neuronal responses to sensory drive as a skew product system; where generalized synchronization requires the CLE of the neuronal system to be negative. However, generalized synchronization is not a complete description of how external states entrain the internal states of self-organizing systems: Entrainment rests upon minimizing free energy that, we conjecture, has an inherent instability. This instability or self-organized critical slowing is due to the fact that internal states with a low free energy are necessarily states with a low free energy curvature. Statistically, this ensures that conditional expectations maintain a conditional indifference or uncertainty that allows for a flexible and veridical representation of hidden states in the world. Dynamically, this low curvature ameliorates dissipation by reducing the (dissipative) update, relative to the (conservative) prediction. In other words, the particular dynamics associated with variational free energy minimization may have a built-in tendency to instability.

It should be noted, that this conjecture deals only with dynamical (gradient descent) minimization of free energy. One could also argue that chaotic itinerancy may be necessary for exploring different conditional expectations to select the one with the smallest free energy. However, it is interesting to note that—even with a deterministic gradient descent—there are reasons to conjecture a tendency to instability. The sort of self-organized instability is closely related to, but is distinct from, chaotic itinerancy and classical self-organized criticality. Chaotic itinerancy deals with itinerant dynamics of deterministic systems that are reciprocally coupled to each other (Tsuda, 2001). Here, we are dealing with systems with a skew product (master-slave) structure. However, it may be that both chaotic itinerancy and critical slowing share the same hallmark; namely, fluctuations of the local Lyapunov exponents around small (near zero) values (Tsuda and Fujii, 2004).

Classical self-organized criticality usually refers to the intermittent behavior of skew product systems in which the drive is constant. This contrasts with the current situation, where we consider the driving system (the environment) to show chaotic itinerancy. In self-organized criticality, one generally sees intermittency with characteristic power laws pertaining to macroscopic behaviors. It would be nice to have a general theory linking the organization of microscopic dynamics in terms of CLE to the macroscopic phenomena studied in self-organized criticality. However, work in this area is generally restricted to specific systems. For example, (Cessac et al., 2001) discuss Lyapunov exponents in the setting of the Zhang model of self-organized criticality. They show that small CLE are associated with energy transport and derive bounds on the principal negative CLE in terms of the energy flux dissipated at the boundaries per unit of time. Using a finite size scaling ansatz for the CLE spectrum, they then relate the scaling exponent to quantities like avalanche size and duration. Whether generalized filtering permits such an analysis is an outstanding question. For the rest of this paper, we will focus on illustrating the more limited phenomena of self-organized critical slowing using simulations of perception.

## Bird song, attractors, and critical slowing

In this section, we illustrate perceptual ignition and critical slowing using neuronal simulations based on the predictive coding scheme of previous sections. Our purpose here is simply to illustrate self-organized instability using numerical simulations: these simulations should be regarded as a proof of principle but should not be taken to indicate that the emergent phenomena are universal or necessary for perceptual inference. In brief, we created sensory stimuli corresponding to bird songs, using a Lorentz attractor with variable control parameters (like the Raleigh number). A synthetic bird then heard the song and used a hierarchical generative model to infer the control parameters and thereby categorise the song. These simulations show how the stimulus induces critical slowing in terms of changes in the CLE of the perceptual dynamics. We then systematically changed the generative model by changing the precision of the motion on hidden states. By repeating the simulations, we could then examine the emergence of critical slowing (averaged over peristimulus time) in relation to changes in variational free energy and categorization performance. Based on the conjecture of the previous section, we anticipated that there will be a regime in which critical slowing was associated with minimum free energy and veridical categorization. In what follows, we describe the stimuli and generative model. We then describe perceptual categorization under optimal prior beliefs about precision and finally characterize the perceptual responses under different (suboptimal) priors.

### A synthetic avian brain

**Figure 4:**
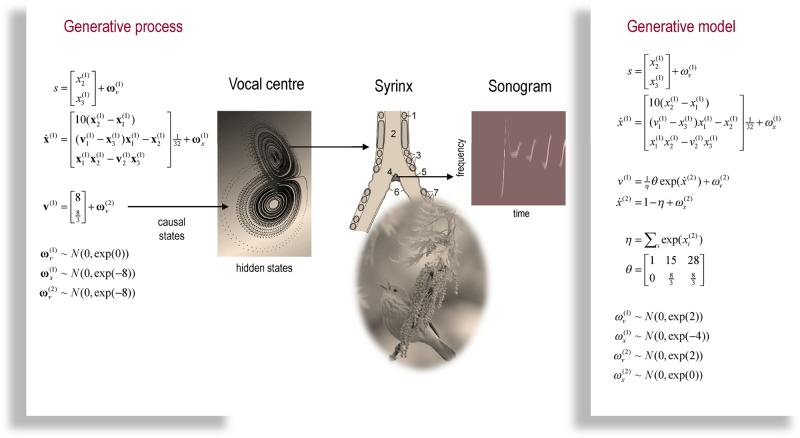
**This is a schematic of stimulus generation and the generative model used for the simulations of bird song perception.** In this setup, the higher vocal centre of a song bird has been modelled with a Lorentz attractor from which two states have been borrowed, to modulate the amplitude and frequency of chirps by its voice box or syrinx. Crucially, the sequence of chirps produced in this way depends upon the shape of the attractor, which is controlled by two hidden causes. This means that we can change the category of song by changing the two hidden causes. This provides a way of generating songs that can be mapped to a point in a two-dimensional perceptual space. The equations on the left describe the production of the stimulus, where the equations of motion for the hidden states correspond to the equations of motion with a Lorentz attractor. These hidden causes were changed smoothly after 32 (16 ms) time bins to transform the attractor from a fixed point attractor (silence) to a chaotic attractor (bird song). The resulting stimulus is shown in sonogram format with time along the x-axis and frequency over the y-axis. The equations on the right constitute the generative model. The generative model is equipped with hidden states at a higher (categorical) level that model the evolution of the hidden causes that determine the attractor manifold for the hidden (attractor) states at the first level. The function generating hidden causes uses a softmax function of the hidden categorical states to select one of three hidden causes. The associated categories of songs correspond to silence, a quasiperiodic song and a chaotic song. The amplitudes of the random fluctuations are determined by their variance or log-precisions and are shown in the lower part of the figure. Using this setup, we can produce some fairly realistic chirps that can be presented to a synthetic bird to see if it can recover the hidden causes and implicitly categorise the song.

The example used here deals with the generation and recognition of bird songs (Zeigler and Marler, 2008; Perl et al., 2011). We imagine that bird songs are produced by two time-varying hidden causes that modulate the frequency and amplitude of vibrations of the syrinx of a song bird (see Figure 4). There has been an extensive modelling effort using attractor models at the biomechanical level to understand the generation of birdsong (Perl et al., 2011). Here we use the attractors at a higher level to provide time-varying control over the resulting sonograms (Kiebel et al., 2009). We drive the syrinx with two states of a Lorenz attractor, one controlling the frequency (between two to five KHz) and the other (after rectification) controlling the amplitude or volume. The parameters of the Lorenz attractor were chosen to generate a short sequence of chirps every second or so. These parameters correspond to hidden causes (**v**^(1)^_1_, **v**^(1)^_2_) that were changed as a function of peristimulus time to switch the attractor into a chaotic state and generate stimuli. Note that these hidden causes have been written in boldface. This is to distinguish them from the hidden causes $\left(v_{1}^{\left(1\right)},v_{2}^{\left(1\right)}\right)$ inferred by the bird hearing the stimuli.

The generative model was equipped with prior beliefs that songs could come in one of three categories; corresponding to three distinct pairs of values for the hidden causes. This was modelled using three hidden states to model the Lorentz attractor dynamics at the first level and three hidden states to model the category of the song at the second level. The hidden causes linking the hidden states at the second level to the first were a weighted mixture of the three pairs of values corresponding to each category of song. The bird was predisposed to infer one and only one category by weighting the control values with a softmax function of the hidden states. This implements a winner-takes-all like behavior and enables us to interpret the softmax function as a probability over the three song categories (softmax probability).

This model of an avian brain may seem a bit contrived or arbitrary; however, it was chosen as a minimal but fairly generic model for perception. It is generic because it has all the ingredients required for perceptual categorization. First, it is hierarchical and accommodates chaotic dynamics in the generation of sensory input. Here, this is modelled as a Lorentz attractor that is subject to small random fluctuations. Second, it has a form that permits categorization of stimuli that extend over (frequency) space and time. In other words, perception, or model inversion maps a continuous, high dimensional sensory trajectory onto a perceptual category or point in some perceptual space. This is implemented by associating each category with a hidden state that induces particular values of the hidden causes. Finally, there is a prior that induces competition or winner-takes-all interactions among categorical representations, implemented using a softmax function. This formal prior (a prior induced by the form of a generative model) simply expresses the prior belief that there is only one cause of any sensory consequence at any time. Together, this provides a generative model based upon highly non-linear and chaotic dynamics that allows competing perceptual hypotheses to explain sensory data.

### Stimulus generation and the generative model

Figure 4 shows a schematic of stimulus generation and the generative model used for categorization. The equations on the left describe the production of the stimulus, where the equations of motion for the hidden states **x**^(1)^∈ ℝ^3^ correspond to the equations of motion with a Lorentz attractor. In all the simulations below, the hidden causes were changed smoothly from **v**^(1)^ = (1, 0) to ${\text{v}}^{\left(1\right)}=\left(28,\frac{8}{3}\right)$ after 32 (16 ms) time bins. This changes the attractor from a fixed point attractor to a chaotic attractor and produces the stimulus onset.

The equations on the right constitute the generative model and have the form of Equation (7). Notice that the generative model is slightly more complicated than the process generating stimuli—it is equipped with hidden states at a higher hierarchical level *x*^(2)^∈ ℝ^3^ that determine the values of the hidden causes, which control the attractor manifold for the hidden states *x*^(1)^∈ ℝ^3^ at the first level. Notice that these hidden states decay uniformly until the sum of their exponentials is equal to one. The function generating hidden causes implements a softmax mixture of three potential values for the hidden causes *v*^(1)^∈ ℝ^3^ encoded in the matrix θ ∈ ℝ^2×3^. The three categories of songs correspond to silence, a quasiperiodic song and a chaotic song. This means that the stimulus changes from silence (the first category) to a chaotic song (the third category). The amplitudes of the random fluctuations are determined by their variance or log-precisions and are shown in the lower part of Figure 4. Given the precise form of the generative model and a stimulus sequence, one can now integrate or solve Equation (8) to simulate neuronal responses encoding conditional expectations and prediction errors.

### Perceptual categorization

**Figure 5:**
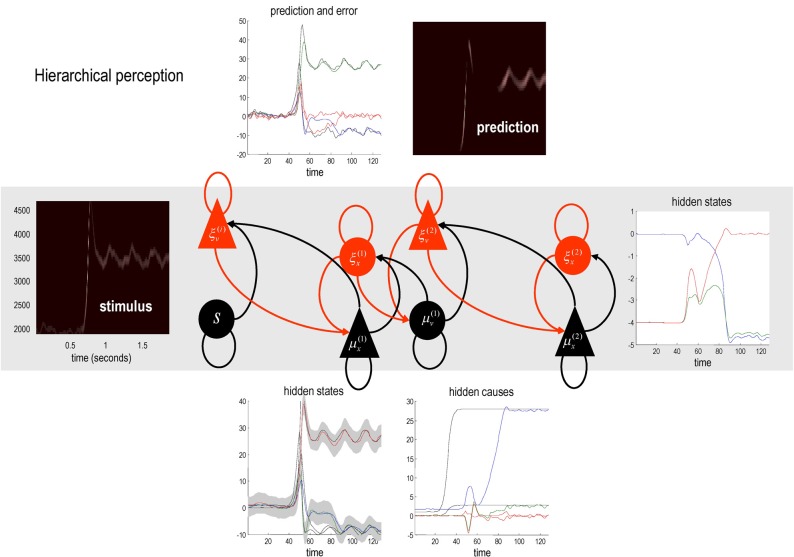
**This reports an example of perceptual categorization following the format of Figure 2.** The panel on the left shows the stimulus in sonogram format, while the corresponding conditional predictions and errors (dotted lines) are shown as functions of time (resp. a sonogram) in the upper left (resp. right) panel. These predictions are based on the expected hidden states at the first level shown on the lower left. The grey areas correspond to 90% conditional confidence intervals. It can be seen that the conditional estimate of the hidden state modulating frequency is estimated reasonably accurately (red Line); however, the corresponding modulation of amplitude takes a couple of chirps before it finds the right level (blue line). This reflects changes in the conditional expectations about hidden causes and the implicit category of the song. The correct category is only inferred after about 80 time bins (red line in the right panel), when expectations of the second level hidden states are driven by ascending prediction errors to their appropriate values.

Figure 5 shows an example of perceptual categorization using the format of Figure 2. The panel on the left shows the stimulus in sonogram format, while the corresponding conditional predictions and errors (dotted lines) are shown as functions of time (resp. a sonogram) in the upper left (resp. right) panel. These predictions are based on the expected hidden states at the first level shown on the lower left. The grey areas correspond to conditional confidence intervals of 90%. It can be seen that the conditional estimate of the hidden state modulating frequency is estimated reasonably accurately (red Line); however, the corresponding modulation of amplitude takes a couple of chirps before it finds the right level (blue line). This reflects changes in the conditional expectations about hidden causes and the implicit category of the song. The correct (third) category is only inferred after about 80 time bins (red line in the right panel), when expectations of the second level hidden states are driven by ascending prediction errors to their appropriate values.

**Figure 6:**
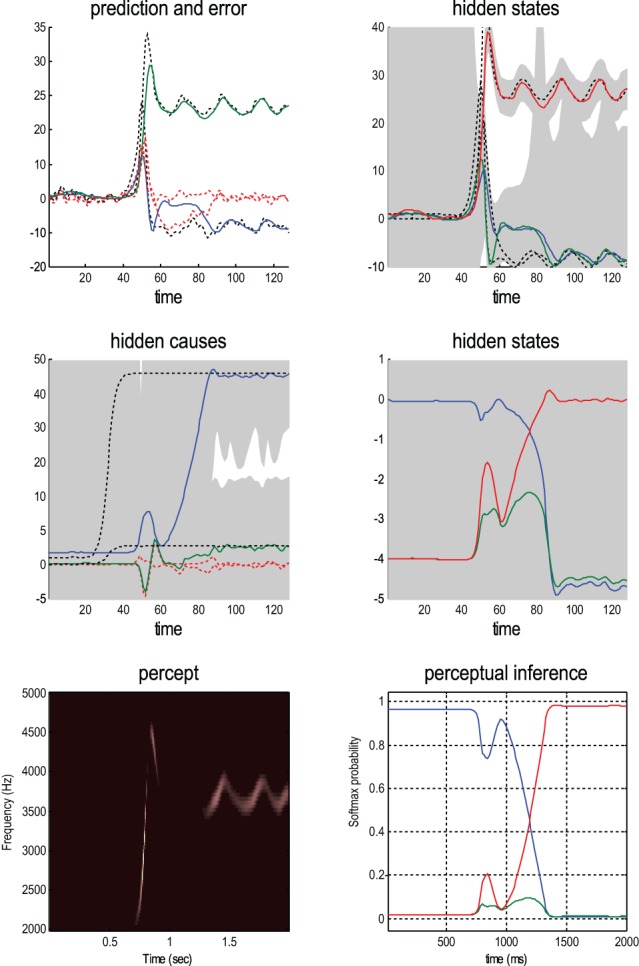
**This shows the same results as in Figure 5 with conditional confidence intervals on all hidden states and causes and the implicit softmax probabilities based on the hidden states at the second level (lower right panel).** These results illustrate switching from the first (silence) to the third (bird song) category (blue and red lines in the lower right panels). This switch occurs after a period of exposure to the new song and enables the stimulus to be predicted more accurately. These dynamics can also be regarded as generalized synchronization between simulated neuronal activity and the true hidden states generating the stimulus.

Figure 6 shows the same results with conditional confidence intervals on all hidden states and causes and the implicit softmax probabilities based on the categorical hidden states at the second level (lower right panel). Note the high degree of uncertainty about the first hidden attractor state, which can only be inferred on the basis of changes (generalized motion) in second and third states that are informed directly by the frequency and amplitude of the stimulus. These results illustrate perceptual ignition of dynamics in higher levels of the hierarchical model that show an almost categorical switch from the first to the third category (from blue to red in the lower right panels). This ignition occurs after a period of exposure to the new song and enables it to be predicted more accurately. These dynamics can also be regarded as a generalized synchronization of simulated neuronal activity with the true hidden states generating the stimulus. So is there any evidence for critical slowing?

### Perceptual instability and switching

**Figure 7:**
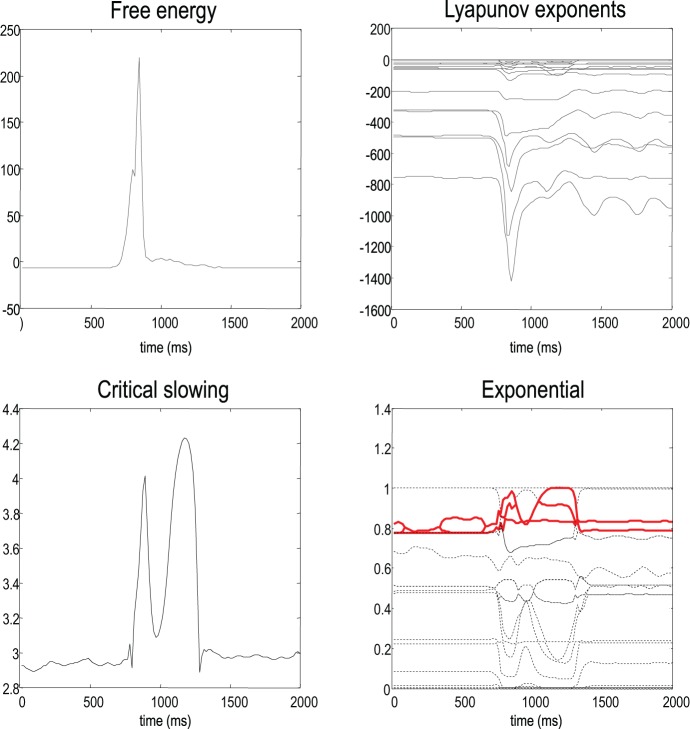
**This shows the evolution of free energy and CLE over peristimulus time.** The upper left panel shows a phasic excess of free energy at the stimulus onset (first chirp or frequency glide). This is quickly resolved by changes in conditional expectations to reduce free energy to prestimulus levels. This reduction changes the Jacobian of the motion of internal states (conditional expectations) and the local conditional Lyapunov exponents (CLE), as shown on the upper right. The lower left panel shows a pronounced critical slowing, as quantified by Equation (17) (using τ = 8 time bins or 128 ms) from stimulus onset to the restoration of minimal free energy. The panels on the right show the underlying changes in the CLE (upper right panel) and their exponentials (lower right panel). The measure of critical slowing is the sum of exponential CLE. It can be seen that several CLE with small negative values move toward zero (highlighted in red). These changes dominate the measure of critical slowing and reflect self-organized instability following stimulus onset—an instability that coincides with the perceptual switch to the correct stimulus category (see previous figure).

Figure 7 shows the evolution of free energy and CLE as a function of peristimulus time. The upper left panel shows a phasic excess of free energy at the stimulus onset (first chirp or frequency glide). This is resolved quickly by changes in conditional expectations to reduce free energy to prestimulus levels. This reduction changes the flow and Jacobian of the conditional expectations and the local CLE as shown on the upper right. Remarkably, there is pronounced critical slowing, as quantified by Equation (17) (using τ = 8 time bins or 128 ms), from the period of stimulus onset to the restoration of minimal free energy. The panels on the right show the underlying changes in the CLE—in their raw form (upper right panel) and their exponentials (lower right panel). The measure of critical slowing is simply the sum of these exponential CLE. It can be seen that many large negative CLE actually decrease their values, suggesting that some subspace of the generalized descent becomes more stable. However, the key change is in the CLE with small negative values, where several move towards zero (highlighted in red). These changes dominate the measure of critical slowing and reflect self-organized instability following stimulus onset—an instability that coincides exactly with the perceptual switch to the correct category of stimulus (see previous figure).

### Perception and critical slowing

The changes described above are over peristimulus time and reflect local CLE. Although we will not present an analysis of global CLE, we can average the local values over the second half of peristimulus time during which the chaotic song is presented. To test our conjecture that free energy minimization and perceptual inference induce critical slowing, we repeated the above simulations while manipulating the (prior beliefs about) precision of the motion of hidden attractor states.

**Figure 8:**
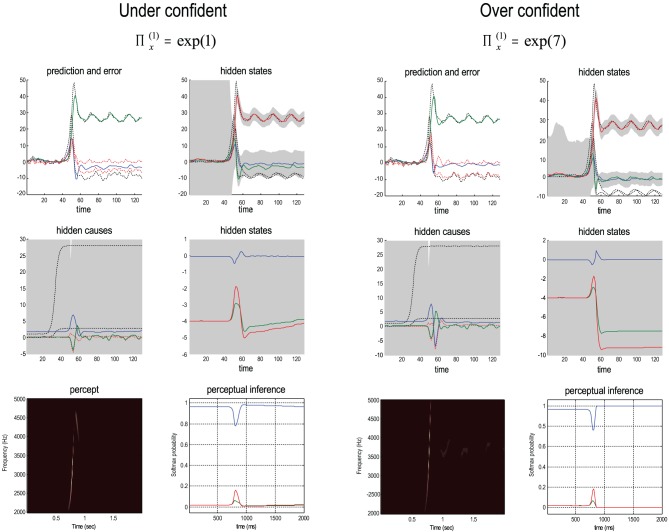
**Failures of perceptual inference illustrated using the same format as Figure 6.** The left panels show the results of decreasing the log precision on the notion of hidden states from 4 to 1; while the right panels show the equivalent results when increasing the log precision from 4 to 7. These simulations represent perceptual categorization with under and over confident beliefs about the motion of hidden attractor states. In both instances, there is a failure of perception of all but the frequency glide at the onset of the song (compare the sonograms in Figure 8 with that in Figure 6). In the under confident condition, there is a slight deviation of predictions about amplitude from baseline (zero) levels—but this is not sufficiently informed by (imprecise) top-down empirical priors to provide a veridical prediction. Conversely, in the over confident condition, the amplitude predictions are impervious to sensory input and reflect top-down prior beliefs that the bird is listening in silence.

Bayes-optimal inference depends upon a delicate balance in the precisions assumed for the random fluctuations at each level of hierarchical models. These prior beliefs are encoded by the log precisions in Equation (8). When intermediate levels are deemed too precise, top-down empirical priors overwhelm sensory evidence, resulting in illusory predictions. Furthermore, they predominate over the less precise prior beliefs at higher levels in the hierarchy. This can lead to false inference and a failure to recognise the high-level causes of sensory inputs. Conversely, when intermediate precisions are too low, the prediction errors from intermediate levels are insufficiently precise to change higher level conditional expectations. This again, can lead to false perception, even if low-level attributes are represented more accurately. These failures of inference are illustrated in Figure 8, using the same format as Figure 6. The left panels show the results of decreasing the log precision on the motion of hidden states from 4 to 1, while the right panels show the equivalent results when increasing the log precision from 4 to 7. These simulations represent perceptual categorization with under and over confident beliefs about the chaotic motion of the hidden attractor states. In both instances, there is a failure of perception of all but the frequency glide at the onset of the song (compare the sonograms in Figure 8 with that in Figure 6). In both cases, this is due to a failure of inference about the hidden categorical states that would normally augment the predictions of hidden attractor states and subsequent sensations. In the under confident condition, there is a slight deviation of predictions about amplitude from baseline (zero) levels—but this is not sufficiently informed by top-down empirical priors to provide a veridical prediction. Conversely, in the over confident condition, the amplitude predictions remain impervious to sensory input and reflect top-down prior beliefs that the bird is listening to silence. Notice the shrinkage in conditional uncertainty about the first hidden attractor state (green line) in the upper right panels. This reflects the increase in precision of the motion of these hidden states.

**Figure 9:**
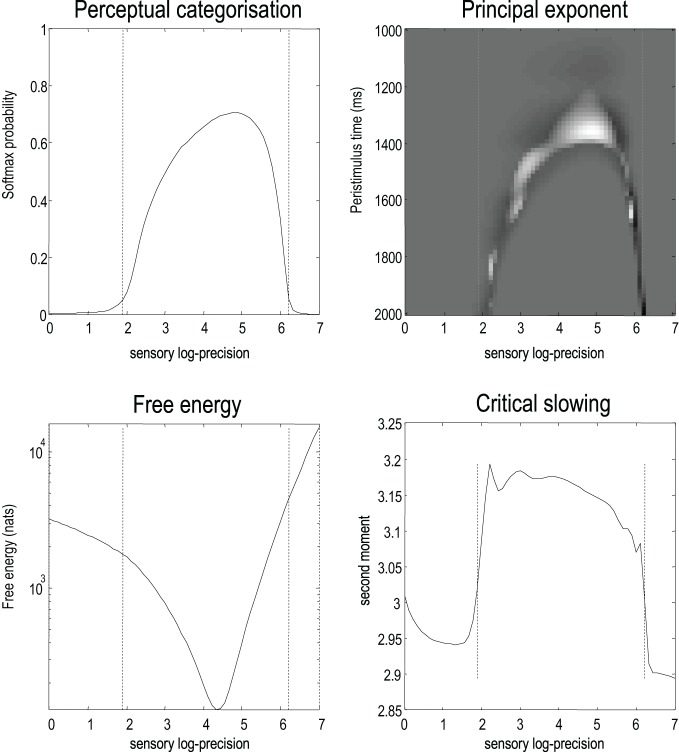
**The upper left panel shows the average probability (following stimulus onset) of correctly identifying a song over 64 values of precision on the motion of hidden attractor states.** The two vertical lines correspond to the onset and offset of nontrivial categorization—a softmax probability of greater than 0.05. The variation in these average probabilities is due to the latency of the perceptual switch to the correct song. This can be seen in the upper right panel that shows the principal CLE in image format as a function of peristimulus time (columns) and precision (rows). It can be seen that the principal CLE shows fluctuations in, and only, in the regime of veridical categorization. Crucially, these fluctuations appear earlier when the categorization probabilities were higher, indicating short latency perceptual switches. The lower left panel shows the time averaged free energy as a function of precision. As one might anticipate, this exhibits a clear minimum around the level of precision that produces the best perceptual categorization. The lower right panel shows a very clear critical slowing in, and only in, the regime of correct categorization. In short, these results are consistent with the conjecture that free energy minimization can induce instability and thereby provide a more responsive representation of hidden states in the world.

Finally, we repeated the above simulations for 64 values of precision on the motion of hidden attractor states from a log precision of zero (a variance of one) to a log precision of seven. At each value, we computed the time average of free energy, the softmax probability of the correct stimulus category and critical slowing. In addition, we recorded the principal local CLE for each simulation. Figure 9 shows the interrelationships among these characterizations: the upper left panel shows the average probability of correctly identifying the song, which ranges from zero in the low and high precision regime, to about 70% in the intermediate regime. The two vertical lines correspond to the onset and offset of nontrivial categorization, with a softmax probability of greater than 0.05. The variation in these average probabilities is due to the latency of the perceptual switch to the correct song. This can be seen in the upper right panel that shows the principal CLE in image format as a function of peristimulus time (columns) and precision (rows). It can be seen that the principal CLE shows fluctuations in, and only, in the regime of veridical categorization. Crucially, these fluctuations appear earlier when the categorization probabilities were higher, indicating short latency perceptual switches. Note that the principal CLE attains positive values for short periods of time. This does not necessarily mean a loss of generalized synchronization; provided the long-term time average is zero or less, when evaluated over long stimulus presentation times. Given that we are looking explicitly at stimulus responses or transients, these positive values could be taken as evidence for transient chaos.

The lower left panel shows the average free energy as a function of precision. As one might anticipate, this exhibits a clear minimum around the level of precision that produces the best perceptual categorization. The key results, from point of view of this paper, are presented in the lower right panel. This shows a very clear critical slowing in, and only in the regime of correct categorization. In short, these results are entirely consistent with the conjecture that free energy minimization induces instability or critical slowing and thereby provides a more veridical representation of hidden states in the world.

## Summary

In summary, these simulations of perceptual transitions affirm the notion that a sensitive response to sensory perturbations from the environment is accompanied by critical slowing of representational dynamics—of the sort that would be predicted by Bayes-optimal perception and the implicit maximum entropy principle. Although we have focused on perception, the imperative to minimize free energy, in the larger setting of active inference, may mean that any self-organizing system that resists a dispersion of its (sensory) states should show the same sort of critical slowing. The perceptual categories used in this paper to illustrate perceptual transitions were very distinct. One might imagine that the role of critical slowing and transitions may become more important when discriminating between more ambiguous stimuli; for example, those used to elicit bistable perception. In future work, we hope to look at bistable perception (binocular rivalry) and revisit our recent work in this area, in terms of critical slowing. In these models, the system works at the border of a Hopf bifurcation, where noise is more efficient in provoking perceptual transitions (Theodoni et al., 2011).

## Conclusion

We have addressed self-organization at a number of levels. First, we have looked at self-organization in terms of the selective sampling of the environment to minimize surprise (free energy) and therefore maintain a homoeostasis in the sense of Ashby (Ashby, 1947). Because surprise is negative log evidence in statistics, free energy minimization can also be understood as accumulating evidence for generative models of the world in a Bayes-optimal fashion. Second, we have considered free energy minimization in self-organizing systems as a dynamical process that performs a (generalized) gradient descent. Statistically speaking, this corresponds to a generalized (Bayesian) filtering or deconvolution that discovers the underlying causes of sensory states. This form of dynamics has the rather curious property of self-destabilization; in the sense that the internal states of a system (like the brain) will seek out regions of low free energy that, by definition, have a low curvature and invite relatively unstable (slow) dynamics. This form of self-organizing instability was demonstrated using neuronal simulations of perceptual categorization and a fairly minimal, but generic generative model. These demonstrations provided an example of Bayes-optimal perceptual categorization that was associated with self-organized instability or critical slowing that may be an integral part of perceptual switching or ignition. Finally, there is an important third level of self-organization that is implicit in the final simulations: at the beginning, we established that the internal states of a self-organizing system will minimize free energy. *This includes posterior beliefs about (estimates of) the precision of random fluctuations*. This means, had we allowed the precision on the motion of hidden attractor states to minimize free energy, it would have found the value that is in the centre of the region showing critical slowing. In other words, that if the system chose the level of uncertainty or confidence in its prior beliefs, it would choose a critical regime. See Figure 9. This is a nice illustration of how self-organization can induce self-organization in subtle and recursive fashion.

### Conflict of interest statement

The authors declare that the research was conducted in the absence of any commercial or financial relationships that could be construed as a potential conflict of interest.

## References

[B1] Abarbanel H. D. Creveling D. R. Jeanne J. M. (2008). Estimation of parameters in nonlinear systems using balanced synchronization. Phys. Rev. E Stat. Nonlin. Soft Matter Phys. 77, 016208 10.1103/PhysRevE.77.016208 18351927

[B2] Ashby W. R. (1947). Principles of the self-organizing dynamic system. J. Gen. Psychol. 37, 125–128 10.1080/00221309.1947.9918144 20270223

[B3] Bak P. Tang C. Wiesenfeld K. (1987). Self-organized criticality: an explanation of 1/f noise. Phys. Rev. Lett. 59, 381–384 10.1103/PhysRevLett.59.381 10035754

[B4] Ballard D. H. Hinton G. E. Sejnowski T. J. (1983). Parallel visual computation. Nature 306, 21–26 6633656 10.1038/306021a0

[B5] Barreto E. Josic K. Morales C. J. Sander E. So P. (2003). The geometry of chaos synchronization. Chaos 13, 151–164 10.1063/1.1512927 12675422

[B6] Beal M. J. (2003). Variational Algorithms for Approximate Bayesian Inference. Ph.D. thesis, University College London, London, UK

[B7] Beggs J. M. Plenz D. (2003). Neuronal avalanches in neocortical circuits. J. Neurosci. 23, 11167–11177 14657176 10.1523/JNEUROSCI.23-35-11167.2003 PMC6741045

[B8] Birkhoff G. D. (1931). Proof of the ergodic theorem. Proc. Natl. Acad. Sci. U.S.A. 17, 656–660 16577406 10.1073/pnas.17.2.656 PMC1076138

[B9] Breakspear M. (2001). Perception of odors by a nonlinear model of the olfactory bulb. Int. J. Neural Syst. 11, 101–124 14632166 10.1142/S0129065701000564

[B10] Breakspear M. (2004). Dynamic connectivity in neural systems - theoretical and empirical considerations. Neuroinformatics 2, 205–225 10.1385/NI:2:2:205 15319517

[B11] Breakspear M. Heitmann S. Daffertshofer A. (2010). Generative models of cortical oscillations: neurobiological implications of the Kuramoto model. Front. Hum. Neurosci. 4:190 10.3389/fnhum.2010.00190 21151358 PMC2995481

[B12] Breakspear M. Stam C. J. (2005). Dynamics of a neural system with a multiscale architecture. Philos. Trans. R. Soc. Lond. B Biol. Sci. 360, 1051–1074 10.1098/rstb.2005.1643 16087448 PMC1854927

[B13] Bressler S. L. Tognoli E. (2006). Operational principles of neurocognitive networks. Int. J. Psychophysiol. 60, 139–148 10.1016/j.ijpsycho.2005.12.008 16490271

[B14] Brown H. Friston K. J. (2012). Free-energy and illusions: the cornsweet effect. Front. Psychol. 3:43 10.3389/fpsyg.2012.00043 22393327 PMC3289982

[B15] Cessac B. Blanchard P. Krüger T. (2001). Lyapunov exponents and transport in the Zhang model of self-organized criticality. Phys. Rev. E Stat. Nonlin. Soft Matter Phys. 64 1 Pt 2, 016133 10.1103/PhysRevE.64.016133 11461357

[B16] Dayan P. Hinton G. E. Neal R. (1995). The Helmholtz machine. Neural Comput. 7, 889–904 7584891 10.1162/neco.1995.7.5.889

[B17] Dehaene S. Changeux J.-P. (2011). Experimental and theoretical approaches to conscious processing. Neuron 70, 200–227 10.1016/j.neuron.2011.03.018 21521609

[B18] Feldman H. Friston K. J. (2010). Attention, uncertainty, and free-energy. Front. Hum. Neurosci. 4:215 10.3389/fnhum.2010.00215 21160551 PMC3001758

[B19] Feldman A. G. Levin M. F. (1995). The origin and use of positional frames of reference in motor control. Behav. Brain Sci. 18, 723–806

[B20] Feynman R. P. (1972). Statistical Mechanics. Reading, MA: Benjamin

[B21] Fisch L. Privman E. Ramot M. Harel M. Nir Y. Kipervasser S. Andelman F. Neufeld M. Y. Kramer U. Fried I. Malach R. (2009). Neural ignition: enhanced activation linked to perceptual awareness in human ventral stream visual cortex. Neuron 64, 562–574 10.1016/j.neuron.2009.11.001 19945397 PMC2854160

[B22] Freeman W. J. (1994). Characterization of state transitions in spatially distributed, chaotic, nonlinear, dynamical systems in cerebral cortex. Integr. Physiol. Behav. Sci. 29, 294–306 7811649 10.1007/BF02691333

[B23] Friston K. (2005). A theory of cortical responses. Philos. Trans. R. Soc. Lond. B Biol. Sci. 360, 815–836 10.1098/rstb.2005.1622 15937014 PMC1569488

[B24] Friston K. (2008). Hierarchical models in the brain. PLoS Comput. Biol. 4:e1000211 10.1371/journal.pcbi.1000211 18989391 PMC2570625

[B25] Friston K. (2009). The free-energy principle: a rough guide to the brain? Trends Cogn. Sci. 13, 293–301 10.1016/j.tics.2009.04.005 19559644

[B26] Friston K. (2010). The free-energy principle: a unified brain theory? Nat. Rev. Neurosci. 11, 127–138 10.1038/nrn2787 20068583

[B27] Friston K. Adams R. Perrinet L. Breakspear M. (2012). Perceptions as hypotheses: saccades as experiments. Front. Psychol. 3:151 10.3389/fpsyg.2012.00151 22654776 PMC3361132

[B29] Friston K. Ao P. (2012). Free-energy, value and attractors. Comput. Math. Methods Med. 2012, 937860 10.1155/2012/937860 22229042 PMC3249597

[B30] Friston K. J. Daunizeau J. Kiebel S. J. (2009). Active inference or reinforcement learning? PLoS ONE 4:e6421 10.1371/journal.pone.0006421 19641614 PMC2713351

[B31] Friston K. J. Daunizeau J. Kilner J. Kiebel S. J. (2010). Action and behavior: a free-energy formulation. Biol. Cybern. 102, 227–260 10.1007/s00422-010-0364-z 20148260

[B32] Friston K. J. Shiner T. FitzGerald T. Galea J. M. Adams R. Brown H. Dolan R. J. Moran R. Stephan K. E. Bestmann S. (2012). Dopamine, affordance and active inference. PLoS Comput. Biol. 8:e1002327 10.1371/journal.pcbi.1002327 22241972 PMC3252266

[B33] Friston K. J. Kiebel S. J. (2009a). Predictive coding under the free-energy principle. Philos. Trans. R. Soc. Lond. B Biol. Sci. 364, 1211–1221 10.1098/rstb.2008.0300 19528002 PMC2666703

[B34] Friston K. Kiebel S. (2009b). Cortical circuits for perceptual inference. Neural Netw. 22, 1093–1104 10.1016/j.neunet.2009.07.023 19635656 PMC2796185

[B35] Friston K. Mattout J. Kilner J. (2011). Action understanding and active inference. Biol. Cybern. 104, 137–160 10.1007/s00422-011-0424-z 21327826 PMC3491875

[B36] Friston K. Stephan K. Li B. Daunizeau J. (2010). Generalised Filtering. Math. Prob. Eng. 2010, 621670

[B37] Ginzburg V. L. Landau L. D. (1950). On the theory of superconductivity. Zh. Eksp. Teor. Fiz. 20, 1064

[B38] Gregory R. L. (1980). Perceptions as hypotheses. Philos. Trans. R. Soc. Lond. B Biol. Sci. 290, 181–197 10.1098/rstb.1980.0090 6106237

[B39] Gros C. (2009). Cognitive computation with autonomously active neural networks: an emerging field. Cogn. Comput. 1, 77–99

[B40] Haken H. (1983). Synergetics: An introduction. Non-equilibrium Phase Transition and Self-organization in Physics, Chemistry and Biology, 3rd Edn Berlin: Springer Verlag

[B41] Helmholtz H. (1866/1962). “Concerning the perceptions in general,” in Treatise on Physiological Optics, 3rd Edn Vol. III, ed Southall J. Trans (New York, NY: Dover).

[B42] Hunt B. Ott E. Yorke J. (1997). Differentiable synchronisation of chaos. Phys. Rev. E 55, 4029–4034

[B43] Ishii S. Yoshida W. Yoshimoto J. (2002). Control of exploitation-exploration meta-parameter in reinforcement learning. Neural Netw. 15, 665–687 10.1016/S0893-6080(02)00056-4 12371519

[B44] Jaynes E. T. (1957). Information theory and statistical mechanics. Phys. Rev. 106, 620–630

[B45] Jirsa V. K. Friedrich R. Haken H. Kelso J. A. (1994). A theoretical model of phase transitions in the human brain. Biol. Cybern. 71, 27–35 8054384 10.1007/BF00198909

[B46] Kaplan J. L. Yorke J. A. (1979). “Chaotic behavior of multidimensional difference equations,” in Functional Differential Equations and Approximations of Fixed Points: Proceedings, eds Peitgen H.-O. Walther H.-O. (Bonn, Berlin: Springer-Verlag), 204.

[B47] Kiebel S. J. Daunizeau J. Friston K. J. (2009). Perception and hierarchical dynamics. Front. Neuroinform. 3:20 10.3389/neuro.11.020.2009 19649171 PMC2718783

[B48] Maturana H. R. Varela F. (1980). “Autopoiesis: the organization of the living,” in Autopoiesis and Cognition, eds Cohen R. S. Wartofsky M. W. (Dordrecht, Netherlands: Reidel).

[B49] Mumford D. (1992). On the computational architecture of the neocortex. II. Biol. Cybern. 66, 241–251 1540675 10.1007/BF00198477

[B50] Namikawa J. (2005). Chaotic itinerancy and power-law residence time distribution in stochastic dynamical systems. Phys. Rev. E 72, 026204 10.1103/PhysRevE.72.026204 16196681

[B51] Nara S. (2003). Can potentially useful dynamics to solve complex problems emerge from constrained chaos and/or chaotic itinerancy? Chaos 13, 1110–1121 10.1063/1.1604251 12946204

[B52] Pasquale V. Massobrio P. Bologna L. L. Chiappalone M. Martinoia S. (2008). Self-organization and neuronal avalanches in networks of dissociated cortical neurons. Neuroscience 153, 1354–1369 10.1016/j.neuroscience.2008.03.050 18448256

[B53] Perl Y. S. Arneodo E. M. Amador A. Goller F. Mindlin G. B. (2011). Reconstruction of physiological instructions from Zebra finch song. Phys. Rev. E 84, 051909 10.1103/PhysRevE.84.051909 22181446 PMC3909473

[B54] Plenz D. Thiagarajan T. C. (2007). The organizing principles of neuronal avalanches: cell assemblies in the cortex? Trends Neurosci. 30, 101–110 10.1016/j.tins.2007.01.005 17275102

[B55] Pyragas K. (1997). Conditional Lyapunov exponents from time series. Phys. Rev. E 56, 5183–5187

[B56] Rabinovich M. Huerta R. Laurent G. (2008). Neuroscience. Transient dynamics for neural processing. Science 321, 48–50 10.1126/science.1155564 18599763

[B57] Rao R. P. Ballard D. H. (1999). Predictive coding in the visual cortex: a functional interpretation of some extra-classical receptive-field effects. Nat. Neurosci. 2, 79–87 10.1038/4580 10195184

[B58] Shew W. L. Yang H. Yu S. Roy R. Plenz D. (2011). Information capacity and transmission are maximized in balanced cortical networks with neuronal avalanches. J. Neurosci. 31, 55–63 10.1523/JNEUROSCI.4637-10.2011 21209189 PMC3082868

[B59] Theodoni P. Panagiotaropoulos T. I. Kapoor V. Logothetis N. K. Deco G. (2011). Cortical microcircuit dynamics mediating binocular rivalry: the role of adaptation in inhibition. Front. Hum. Neurosci. 5:145 10.3389/fnhum.2011.00145 22164140 PMC3224982

[B60] Tschacher W. Haken H. (2007). Intentionality in non-equilibrium systems? The functional aspects of self-organised pattern formation. New Ideas Psychol. 25, 1–15

[B61] Tsuda I. (2001). Toward an interpretation of dynamic neural activity in terms of chaotic dynamical systems. Behav. Brain Sci. 24, 793–810 12239890 10.1017/s0140525x01000097

[B62] Tsuda I. Fujii H. (2004). A complex systems approach to an interpretation of dynamic brain activity I: chaotic itinerancy can provide a mathematical basis for information processing in cortical transitory and nonstationary dynamics. LNCS 3146, 109–128

[B63] Tyukin I. Tyukina T. van Leeuwen C. (2009). Invariant template matching in systems with spatiotemporal coding: a matter of instability. Neural Netw. 22, 425–449 10.1016/j.neunet.2009.01.014 19264447

[B64] Tyukin I. van Leeuwen C. Prokhorov D. (2003). Parameter estimation of sigmoid superpositions: dynamical system approach. Neural Comput. 15, 2419–2455 10.1162/089976603322362428 14511528

[B65] van Leeuwen C. (2008). Chaos breeds autonomy: connectionist design between bias and baby-sitting. Cogn. Process. 9, 83–92 10.1007/s10339-007-0193-8 17924155

[B66] Werner G. (2007). Brain dynamics across levels of organization. J. Physiol. Paris 4–6, 273–279 10.1016/j.jphysparis.2007.12.001 18267356

[B67] Yuille A. Kersten D. (2006). Vision as Bayesian inference: analysis by synthesis? Trends Cogn. Sci. 10, 301–308 10.1016/j.tics.2006.05.002 16784882

[B68] Zeigler P. Marler P. (2008). Neuroscience of Birdsong. Cambridge, MA: Cambridge University Press

